# Eluding the illusion? Schizophrenia, dopamine and the McGurk effect

**DOI:** 10.3389/fnhum.2014.00565

**Published:** 2014-08-05

**Authors:** Thomas P. White, Rebekah L. Wigton, Dan W. Joyce, Tracy Bobin, Christian Ferragamo, Nisha Wasim, Stephen Lisk, Sukhwinder S. Shergill

**Affiliations:** Department of Psychosis Studies, Institute of Psychiatry, King's College LondonLondon, UK

**Keywords:** schizophrenia, dopamine, McGurk effect, multisensory integration, probabilistic inference

## Abstract

Perceptions are inherently probabilistic; and can be potentially manipulated to induce illusory experience by the presentation of ambiguous or improbable evidence under selective (spatio-temporal) constraints. Accordingly, perception of the McGurk effect, by which individuals misperceive specific incongruent visual and auditory vocal cues, rests upon effective probabilistic inference. Here, we report findings from a behavioral investigation of illusory perception and related metacognitive evaluation during audiovisual integration, conducted in individuals with schizophrenia (*n* = 30) and control subjects (*n* = 24) matched in terms of age, sex, handedness and parental occupation. Controls additionally performed the task after an oral dose of amisulpride (400 mg). Individuals with schizophrenia were observed to exhibit illusory perception less frequently than controls, despite non-significant differences in perceptual performance during control conditions. Furthermore, older individuals with schizophrenia exhibited reduced rates of illusory perception. Subsequent analysis revealed a robust inverse relationship between illness chronicity and the illusory perception rate in this group. Controls demonstrated non-significant modulation of perception by amisulpride; amisulpride was, however, found to elicit increases in subjective confidence in perceptual performance. Overall, these findings are consistent with the idea that impairments in probabilistic inference are exhibited in schizophrenia and exacerbated by illness chronicity. The latter suggests that associated processes are a potentially worthwhile target for therapeutic intervention.

## Introduction

Cross-modal cue integration is adaptively advantageous given the likelihood that real-world events perturb multiple sensory systems; and is sensible from a computational standpoint, as uncertainty in one modality can be reduced by combining with signals from others, increasing the overall precision of a sensory percept. However, perceptual binding is not straightforward because sensory signals vary continuously, instantaneously and unpredictably. Related inferences are therefore necessarily probabilistic. As inter-sensory inconsistencies are likely abundant, perceptual decisions should take into account stimulus-specific uncertainty. Indeed, humans have been consistently shown to fuse multisensory data in a manner that reflects the relative reliability of the information received in line with canonical ideal-observer models including Bayesian decision theory and maximum-likelihood models (Green and Swets, 1966; Ernst and Banks, 2002; van Beers et al., 2002; Alais and Burr, 2004).

The Bayesian perspective provides a general inferential schema to understand brain function. With respect to perception, our experiences can be understood as the most likely explanation of the world (determined via posterior probability distributions), with these inferences biased by our own expectations (or priors reflecting past inferences) as well as data from available stimuli (the conditional likelihood of the data given a model). Critically, these computations lead to updated posterior probability distributions about the world which then serve to adjust future expectations. The fundamental units are prediction error (on expected sensory input) and expected value (of actions executed in the environment), that can be modeled as free energy in a general optimization theory of perception and action (Feldman and Friston, 2010; Friston, 2010).

Speech perception presents us with two illusory phenomena whose occurrence illustrates the role of Bayesian inferences in multisensory fusion. Firstly, the ventriloquist illusion (that an inanimate puppet is speaking) occurs when a ventriloquist produces speech sounds while simultaneously moving a puppet's lips instead of his/her own. This robust effect denotes inaccurate source localization on account of the temporal proximity of the ventriloquist's speech and the puppet's lip movements. The illusory percept can be understood as the consequence of the expectation that multisensory stimuli coincident within specific spatio-temporal limits emanate from a common source, as is supported by the finding that the strength of the ventriloquism effect decreases as spatial and temporal disparity increase (Wallace et al., 2004). The observation that the ventriloquism percept can be increased when stimuli are more spatio-temporally coincident (Radeau, 1994) implies dynamic updating of top-down priors as previous conceptions are revised. Furthermore, the elevated weighting of visual localization cues can be explained by the modality-appropriateness hypothesis that weighting adjustments reflect our resolving capabilities within each sensory modality (Welch and Warren, 1980). Thus, the illusory interpretation is judged the most likely (and therefore optimal) explanation but is erroneous for the idiosyncratic and ambiguous conditions manipulated by the ventriloquist. This notion of illusory experience as a Bayes-optimal inference is elegantly formalized elsewhere (Brown and Friston, 2012).

Another illusory (yet optimal) inference during speech perception was first reported by McGurk and MacDonald (1976), who found that when viewing edited movie clips of an actor articulating one syllable in synchronization with the soundtrack of other (specific) syllables, individuals often perceive a syllable incongruent with either the visual or auditory input (McGurk and MacDonald, 1976). For example, a visual /ga/ with an auditory /ba/ is perceived as /da/; and a visual /ka/ with an auditory /pa/ elicits the perception of /ta/. This perceptual effect can be understood, similarly to the ventriloquist illusion, as reflecting the most plausible auditory percept under the assumption of a common signal source (a single articulating agent), when spatio-temporal multi-modal constraints are met.

These examples suggest that priors are estimated in multiple, hierarchically-arranged contexts: there is a general prior that lip movements and sounds usually come from the same person, which has the effect of inducing integration of visual and auditory information into a single percept; furthermore, priors pertaining to information presented in one modality (e.g., vision) have the potential to influence interpretation of data presented in another modality (e.g., audition); and particularly relevantly to the McGurk effect, further priors dictate the extent to which particular multimodal confluences shape perception. These putative priors accordingly provide explanation for illusions being systematically associated with certain audio-visual combinations but not others. If visual and auditory data are presented simultaneously and are therefore more likely to emanate from a common source (than contradictory independent sources), and their concurrent posterior probability distributions overlap with those of another explanation of the data, then favoring that alternative explanation is probabilistically advantageous. By contrast, if an aspect of the auditory or visual data is more distinct, its posterior distribution will be more precisely defined (i.e., there will be reduced variance around its maximum likelihood estimate), and that cue will have increased weight in the perceptual decision, which may in turn over-ride the prior that the inputs are commonly derived.

Several core features of schizophrenia can be understood in terms of defective Bayesian processes (Corlett et al., 2009; Fletcher and Frith, 2009). Abnormal interactions between prior beliefs and presented sensations plausibly hinder attempts to prioritize, learn about and understand the world (Gray et al., 1991); more dramatically, over-powering priors may cause the experience of a non-existent stimulus (hallucination), or abnormal bottom-up signaling may instill a conventionally-irrelevant event with enhanced significance (laying the foundation for a delusional belief) (Fletcher and Frith, 2009; Joyce et al., 2013). Pervasive deficits in information processing—including those relating to multisensory integration—further intimate impaired Bayesian inferences in the disorder (Heinz and Schlagenhauf, 2010; Shergill et al., 2014). Most relevantly here are previous observations that individuals with schizophrenia are less likely to perceive the McGurk effect (de Gelder et al., 2005; Pearl et al., 2009), although it is noteworthy that preserved multisensory fusion also been reported (Myslobodsky et al., 1992; Martin et al., 2013).

Bayesian models of the world require two fundamental inferences: updating a representation of the state of the world; and a judgment about the associated level of uncertainty (Friston et al., 2012). Dopamine has been proposed to play a role in the top-down specification of priors (Scott et al., 2007; Corlett et al., 2009), coding uncertainty by means of synaptic gain control (Friston et al., 2012). Gain—the rate at which neuronal output changes in relation to the strength of driving inputs—fundamentally promotes the amplification of contextually-relevant signals and the consequent reduction of noise in the inputs, which in turn imparts advantages for optimizing signal detection. This can play diverse computational roles; but in terms of multisensory integration is thought to promote appropriate binding by synchronizing event-related oscillatory activity through the coordinated action of fast-spiking inhibitory interneurons (Phillips and Silverstein, 2013).

Kapur's (2003) notion that delusions are formed when spontaneous, phasic striatal dopamine firing imbues salience to contextually-irrelevant stimuli presents a mechanistic link between Bayesian cognition and schizophrenic symptomatology. Individuals with schizophrenia exhibit an elevated baseline dopaminergic state (Laruelle et al., 1996), but reduced event-related, context-relevant dopamine firing (Kapur, 2003; Heinz and Schlagenhauf, 2010). The latter supports the notion that abnormal gain control and prior specification explain the pervasive sensory and decision-making deficits in the disorder (Phillips and Silverstein, 2013). This pathological firing can explain reduced susceptibility to illusory multisensory stimuli in schizophrenia since inconsistent dopamine-coded, event-specific precision (Friston, 2005) potentially reduces the likelihood of appropriately binding encountered stimuli from discrete modalities. Relatedly, it has been shown that dopamine agonists facilitate visual discriminative performance and enhance perceived sensory acuity; and that this improvement is associated with greater subjective feelings of event-related confidence (Fillmore et al., 2005; Lou et al., 2011). The investigation of the relationship between multisensory integration and metacognitive confidence judgments in schizophrenia provides an accessible means of increasing our understanding of the associated role of dopaminergic dysregulation. However, investigating dopaminergic modulation in association with pathology is associated with numerous confounds. Pharmacological manipulation of dopaminergic processing in healthy individuals provides an alternative investigative model.

Central to accounts of perception as probabilistic inference is their dynamic updating, which occurs on multiple timescales. In light of modality-specific developmental and degenerative changes in sensory acuity through the lifespan (Gordon-Salant, 2005), the modality-appropriateness hypothesis, and the role of cumulative experience, age is a likely moderator of the efficiency of multisensory integration. McGurk perception has been previously reported to vary with age, with older adults more likely to experience the McGurk effect than younger adults (Setti et al., 2013). Given characteristic developmental disruption in schizophrenia (Murray and Lewis, 1987), age may also differentially affect multisensory integration in individuals with schizophrenia. This is intimated by the findings that significant differences in illusory McGurk perception have been previously observed in adolescents but not older individuals (Pearl et al., 2009).

The primary aim of this work was to quantify audio-visual performance and related metacognitive judgments to test four explicit hypotheses. First, lower rates of illusory perception were predicted in individuals with schizophrenia as compared with healthy individuals. This prediction was made on account of the converging lines of evidence suggesting perceptual aberrance, disturbances to probabilistic inference, and previous abnormalities in multisensory fusion in schizophrenia (Heinz and Schlagenhauf, 2010; Phillips and Silverstein, 2013), and the notion that illusory perception represents the optimal probabilistic inference. Second, and complementarily, on account of a putative reduction in the acuity of multisensory integration in association with reduced dopaminergic activity, it was hypothesized that illusory McGurk perception would be reduced in healthy individuals following pharmacologically-induced dopamine blockade. In light of the idea that probabilistic inferences are dynamically updated by experience, it was predicted that schizophrenia-related abnormalities would increase in line with illness chronicity. Finally, the influence of illness, drug and age on metacognitive evaluation of audio-visual performance during the McGurk task was assessed to test the fourth hypothesis that these factors shape higher-order subjective experiences associated with multisensory fusion in addition to the perceptual experiences themselves.

## Methods

### Participants

Thirty right-handed individuals satisfying DSM-IV criteria for schizophrenia and 24 right-handed control subjects group-matched for age, sex, handedness and socioeconomic background assessed on the basis of parental occupation using the National Statistics Socio-Economic Classification (Rose and Pevalin, 2001), were recruited to take part in this behavioral study. Intelligence quotient (IQ) was measured using the two-item Wechsler Abbreviated Scale of Intelligence (WASI) (Wechsler, 1999) and was lower in the schizophrenia group as compared with the healthy group [*t*_(51)_ = 3.11, *p* = 0.003]. Table 1A presents demographic characteristics of the study sample. Ethical approval was provided by Central London Research and Ethics Committee 1. All participants provided informed written consent and were given a monetary inconvenience allowance for their participation in the study.

**Table 1:** **Sample details**.

**Measure**	**Group**		
	**Schizophrenia group (*n* = 30)**	**Healthy group (*n* = 24)**	**Between-group difference**
**(A) GROUP MEAN DEMOGRAPHIC DETAILS. BRACKETED VALUES DENOTE STANDARD DEVIATION**			
Age (years)	39.03 (8.60)	36.67 (8.97)	*t*_(51)_ = 0.98, *p* = 0.332
	Range: 25–54	Range: 22–50	
Sex (male/female)	27/3	21/3	χ^2^ (1, *N* = 53) = 0.08, *p* = 0.771
Parental occupation (NS-SEC)	3.10 (1.70)	2.50 (1.67)	*t*_(51)_ = 1.30, *p* = 0.200
Intelligence quotient (WASI)	95.62 (13.68)	108.21 (15.80)	*t*_(51)_ = 3.11, *p* = 0.003
Chlorpromazine equivalent doses (mg/day)	463.25 (330.62)		
	Range: 133–1100		
**(B) MEAN SCHIZOPHRENIA GROUP PANSS SCORES. BRACKETED VALUES DENOTE STANDARD DEVIATION**			
Positive	14.32 (4.83)		
Negative	18.96 (5.90)		
General	30.59 (7.09)		

NS-SEC, National Statistics Socio-economic Classification (Rose and Pevalin, 2001); WASI, Wechsler Abbreviated Scale of Intelligence (Wechsler, 1999); PANSS, Positive and negative syndrome scale for schizophrenia (Kay et al., 1987).

Diagnosis of schizophrenia was confirmed by assessment of clinical case notes and endorsement of suitability by each individual's consultant psychiatrist. Onset of illness was also established by clinical case notes. Patients were excluded if presenting evidence of comorbid axis 1 diagnosis, significant medical illness or an IQ < 85. Symptom severity and classification were assessed in the schizophrenia group using the Positive and Negative Syndrome Scale (PANSS) for schizophrenia (Kay et al., 1987). Table 1B presents a summary of the related symptom characteristics of the schizophrenia group. These individuals took part in the study 13.9 ± 8.7 (mean ± standard deviation) years after illness onset.

Twenty nine of the schizophrenia group were medicated at time of study. Twenty six of these were prescribed atypical anti-psychotic medications [olanzapine (*n* = 10), risperidone (*n* = 3), aripirazole (*n* = 2), quetiapine (*n* = 1), clozapine (*n* = 8), amisulpride (*n* = 2), and three typical anti-psychotic medications [zuclopenthixol (*n* = 2), haloperidol (*n* = 1)] at time of participation. Chlorpromazine equivalent doses of prescribed antipsychotic medications were calculated for each individual with schizophrenia using conversion tables (Woods, 2003).

Healthy volunteers were recruited by local poster advertisement. Respondents were excluded from study if they: reported a personal history of psychiatric or neurological illness; exhibited a major current physical illness; demonstrated an IQ < 85; or had a history of psychotic illness in a first-degree relative. Healthy individuals provided negative urine drug-screens at the beginning of each experimental day. All participants reported normal hearing and normal or corrected-to-normal vision.

### Experimental procedure

In order to investigate the principal aims of this study, complementary within- and between-subjects procedures were employed. Effects of pharmacological manipulation on multisensory integration were tested in a within-subjects investigation of the healthy individuals. Healthy individuals underwent procedurally identical dosing and behavioral-task regimes on two testing days separated by 10.10 ± 5.58 days. During one session they received 400 mg amisulpride before performing the multisensory integration task and during the other they received a placebo. The order of these sessions was counter-balanced. (Four healthy individuals only took part in one experimental session thus precluding investigation of drug effects in these individuals. However, these individuals are included in the between-subjects analyses described below where appropriate.)

The investigation of illness effects took place in the context of a between-subjects design, which involved comparing the task performance of healthy individuals after placebo with the performance of individuals with schizophrenia after no dosing supplementary to their currently-prescribed medication. It was not an objective of this work to investigate the effects of single-dose medications in the schizophrenia group as interactions between single-dose medications, long-term medication regimes and underlying pathology are likely to be non-trivial and difficult to dissociate. The decision to refrain from dosing the schizophrenia group with placebo was based on the rationale that a potential subjective difference arises after being told that a drug is a placebo as compared with being told that it is one of several medications, one of which is a placebo.

This study was part of a larger neuroimaging investigation contrasting the effects of dopamine agonism (by means of a single 0.25 mg oral dose of ropinirole) and antagonism (by single amisulpride dosing). Amisulpride is a 2-methoxybenzamide derivative with high affinity for dopamine D_2_ and D_3_ receptors, which is widely prescribed as an antipsychotic medication. It is rapidly absorbed, exhibiting two distinct plasma concentration peaks, which are typically around 1 and 4 h post-administration (Kudris et al., 2011). Data were not collected for multisensory perceptual performance after ropinirole on account of its shorter plasma concentration peak which rendered performance of the current task incompatible with other (neuroimaging-related) study requirements. It is envisaged that the major findings from the neuroimaging component of the study will be published elsewhere.

Dosing took place 1 h prior to experimental task performance with timing in line with previously reported initial peak in circulating concentrations of amisulpride following oral ingestion.

The double-blind dosing procedure was identical for both sessions. The participant was given a small plastic cup containing an encapsulated tablet of either 400 mg amisulpride or a placebo, and instructed to empty the cup directly into his/her mouth before swallowing with water from another plastic cup. This approach was employed to minimize manual contact with either tablet to reduce the likelihood of the participant identifying the tablet, although it is emphasized that during pharmacist-conducted drug preparation attempts were made to ensure qualitative similarity between the tablets.

#### Behavioral task

The audio-visual task was performed in a quiet testing room at the Institute of Psychiatry. Participants sat comfortably in front of a laptop computer with a 15-inch liquid crystal display monitor, and a supplementary mouse. They wore binaural, noise-canceling headphones through which the auditory stimuli were presented. The task was run using the Cogent toolbox (version 1.30; http://www.vislab.ucl.ac.uk) within Matlab (version 7.10; Mathworks, USA).

Participants were informed that they would view an actor articulating a syllable and both visual and auditory cues would be presented to them; and were requested to report the syllable spoken by the actor. After presentation of this multisensory stimulus, responses were made by button press on the laptop keyboard. After each report, the participants were additionally required to judge their subjective confidence in relation to this syllable judgment using a visual analog scale (VAS) ranging from 0 (not confident) to 10 (confident). VAS reports were made by placing the laptop cursor along this VAS.

A male actor, unknown to the participants, was digitally recorded while articulating the phonemes /ba/, /ga/, /pa/ and /ka/ in a neutral manner. This study used meaningless syllables to reduce the impact of higher-order semantic factors which could otherwise underlie between-group performance differences. During recording the actor stood in front of a plain background. The resulting data files were split into visual and audio components and recombined in eight different combinations, ensuring synchronization between lip movement and sound. Auditory and visual components were paired to make eight different conditions of three general classes: (1) congruent stimuli, which were: congruent /ba/ (visual: /ba/, auditory: /ba/); congruent /ga/ (visual: /ga/, auditory: /ga/); and congruent /pa/ (visual: /pa/, auditory: /pa/); congruent /ka/ (visual: /ka/, auditory: /ka/); (2) incongruent McGurk-inducing stimuli, which were: McGurk /da/ (visual: /ga/, auditory /ba/, McGurk perception: /da/); and McGurk /ta/ (visual: /ka/, auditory: /pa/, McGurk perception: /ta/); and (3) incongruent stimuli that do not produce the McGurk effect: incongruent /ga/ (visual: /ba/, auditory: /ga/, common perception: /ga/); and incongruent /ka/ (visual: /pa/, auditory: /ka/, common perception /ka/).

Each trial comprised: the presentation of a multisensory stimulus; a visually-presented question asking the participant which syllable the actor had spoken from a constant list of alternatives (/ba/, /ga/, /da/, /pa/, /ka/ and /ta/); a related button-press choice; a visually-presented question asking the participant to rate their confidence in their preceding judgment along with a VAS depicting variable confidence; and a point-and-click response to denote confidence in the button-press choice. The response to denote syllable choice comprised typing the letters of the perceived syllable on a standard keyboard (for example, “b” and “a” for /ba/). The response to denote confidence comprised moving a screen cursor and clicking via a mouse. All button responses were made by the participants.

The task involved presentation of a total of 40 McGurk clips, 40 (non-McGurk) incongruent clips and 80 congruent control clips. Progression through the task was participant paced with the task additionally split into 20-clip segments.

### Statistical analysis

In-house scripts run in Matlab (version 7.10; Mathworks, USA) were used to extract behavioral parameters and these data were exported to SPSS (version 21; SPSS Inc, USA) for statistical appraisal. Trials in which the participant entered an incomplete or incomprehensible response were considered invalid and therefore discarded. Individuals failing to identify 75% of control stimuli were excluded from further analysis. As a result, the data from one individual with schizophrenia were omitted.

The McGurk_*illusory*_ rate represents the propensity of an individual to experience the McGurk effect for appropriate stimuli; and was calculated by dividing the number of McGurk responses by the number of valid McGurk trials and multiplying this figure by 100. Illusory responses were considered optimal during McGurk trials under the rationale that they are the most likely explanation under the assumption of a single articulating agent (within spatio-temporal constraints). By contrast, auditorily-governed responses were deemed optimal during incongruent non-McGurk trials under the rationale that people favor auditory cues over visual cues when judging speech. As such, the Incongruent_*optimal*_ rate represented the number of responses signifying perception of the auditory stimulus during incongruent non-McGurk trials by the number of valid incongruent non-McGurk trials and multiplying this figure by 100. Perception in line with congruent auditory and visual evidence was considered optimal during control trials. The Control_*optimal*_ rate was therefore calculated by dividing the number of responses compatible with the multisensory stimuli during control trials by the number of valid control trials and multiplying this figure by 100.

In addition, the McGurk_*visual*_ rate was calculated for each session by dividing the number of McGurk trials in which the response matched the visual stimulus by the number of valid McGurk trials with a non-illusory response and multiplying this figure by 100. Similarly, the McGurk_*auditory*_ rate was calculated for each session by dividing the number of McGurk trials in which the response matched the auditory stimulus by the number of valid McGurk trials with a non-illusory response and multiplying this figure by 100. The rationale for constraining these measures to the non-illusory trials was to control for hypothesized between-group differences in the McGurk_*illusory*_ rate.

#### Investigation of multisensory perception in schizophrenia

A general linear model (GLM) was estimated as the primary analysis to investigate whether audio-visual integration differed in individuals with schizophrenia as compared with healthy individuals, and including McGurk_*illusory*_ rate as the dependent variable, study group (healthy/schizophrenia) as a between-subjects factor and age as a covariate. The main effects of group, age and the interaction of these factors were investigated. The GLM effects are reported using the likelihood ratio χ^2^ statistic as related coefficients are less susceptible to inflation when large (in contrast to the alternative, Wald-statistic values) (Menard, 1995). It was considered worthwhile—on the basis of the results of these analyses—to investigate the relationship between illness duration and the McGurk_*illusory*_ rate in the schizophrenia group. To test whether illness duration predicted task performance, a linear regression was conducted on the schizophrenia group data using illness duration (in years) as the independent variable and the McGurk_*illusory*_ rate as the dependent variable.

The incorporation of incongruent non-McGurk stimuli and control stimuli in the current task permitted investigation of response rates to these stimuli in further GLMs. This in turn enabled the dissociation of illness-related abnormalities in multisensory fusion from other aspects of task performance. Significance thresholds for the tests that beta coefficients significantly differed from zero in these three GLMs were Bonferroni-corrected to 0.017. However, since the principal analysis focused on perception of McGurk stimuli nominally significant effects are additionally reported in relation to this analysis.

Two further GLMs were estimated to investigate non-illusory perception following McGurk stimuli. In these the McGurk_*visual*_ and McGurk_*auditory*_ rates were included as the respective dependent variables; again, the main and interaction effects of age and study group were investigated. To investigate whether there was any relationship between these variables and susceptibility to the McGurk effect, Pearson's test was used to assess correlation between the subject-specific McGurk_*illusory*_ rate and McGurk_*visual*_ and McGurk_*auditory*_ rates for each study group independently.

Exploratory bivariate correlation analyses were conducted using Spearman's test and assessing the relationships between the rates of probabilistically-optimal performance for the three trial types and positive, negative and general symptoms subscales rated with the PANSS interview (Kay et al., 1987). To reflect the nine tests conducted, the critical α-threshold was Bonferroni corrected to 0.006. However, nominally significant associations are reported for illustration. Pearson's test was additionally used to investigate the relationship between IQ and the McGurk_*illusory*_ rate for the sample as a whole and each study group individually.

To investigate whether antipsychotic medication affected task performance, Pearson's test was used to assess the relationships between chlorpromazine equivalent doses and all performance indices calculated as described above for the schizophrenia group.

#### Investigation of drug effects

Three, independent 1 × 2 repeated-measures analysis of variance (ANOVA) tests were conducted using healthy-group data from the amisulpride and placebo sessions to test the within-subject effects of drug on the probabilistically-optimal response rate. These analyses therefore included the individual-specific McGurk_*illusory*_ rate, Incongruent_*optimal*_ rate, and the Control_*optimal*_ rate calculated as above. Age was included as a covariate in these analyses to permit investigation of its effects and their interaction with drug effects.

To investigate whether the McGurk_*visual*_ or McGurk_*auditory*_ rates differed by drug condition, age or the interaction of these factors, two repeated-measures ANOVA tests were conducted with drug as a within-subjects factor and age as a covariate.

#### Investigation of modulation of metacognition

Mean overall confidence ratings were calculated for the three trial types for each individual. In addition, mean confidence ratings were computed for each trial type dissociating optimal-response and other-response trials.

Two 1 × 3 repeated-measures ANOVA tests were carried out to assess whether these ratings differed between individuals with schizophrenia and healthy individuals: (1) mean overall confidence ratings during the three trial types were included as within-subject levels (to permit evaluation of the within-subjects effect of condition), study group was included as a between-subjects factor, and age as a covariate; and (2) mean confidence ratings for the three trial types during trials with optimal perceptual responses were included as within-subject levels, with the corresponding covariate, within- and between-subject factors assessed. It was not considered valid to compare directly confidence ratings in association with optimal and other responses on account of the small proportion of healthy individuals exhibiting consistently suboptimal performance during incongruent non-McGurk trials (*n* = 3).

To investigate the impact of amisulpride on the magnitude of confidence ratings and their relation to perceptual performance two further analyses were performed: (1) a 2 × 3 repeated-measures ANOVA test to examine the within-subject factors of drug and condition-type on mean confidence ratings, and including age as a covarying factor; and (2) a 2 × 3 repeated-measures ANOVA test to assess the within-subject factors of drug and condition-type on mean confidence ratings when the participant gave the optimal answer. This latter analysis also included age as a covariate.

A paired-samples *t*-test was conducted to test the primary hypothesis that amisulpride enhances confidence ratings during McGurk perception. Further, exploratory *t*-tests were conducted to evaluate drug effects on the other aspects of metacognitive performance evaluated during the experimental task. When assessing significance in these latter multiple tests the critical significance threshold was Bonferroni corrected to 0.006.

Effect sizes were calculated using Pearson's correlation coefficient, *r*, on account of the applicability of this approach to diverse test statistics (Rosenthal and DiMatteo, 2001), and are provided for all reported effects.

## Results

### Schizophrenia-related disturbances to audio-visual integration

**Figure 1:**
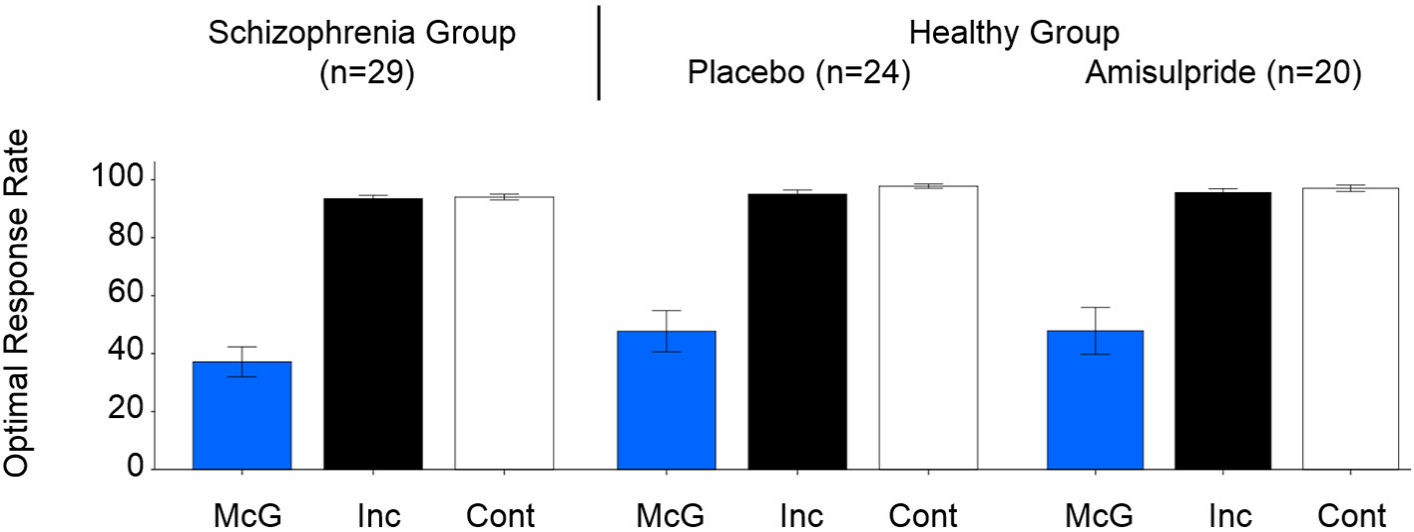
**Group-averaged perceptual performance for the three condition types, for the schizophrenia group (left), and the healthy group after placebo (center) and after amisulpride (right)**. Error bars signify standard errors of the means. McG, McGurk trials; Inc, incongruent non-McGurk trials, Cont, control congruent trials.

**Figure 2:**
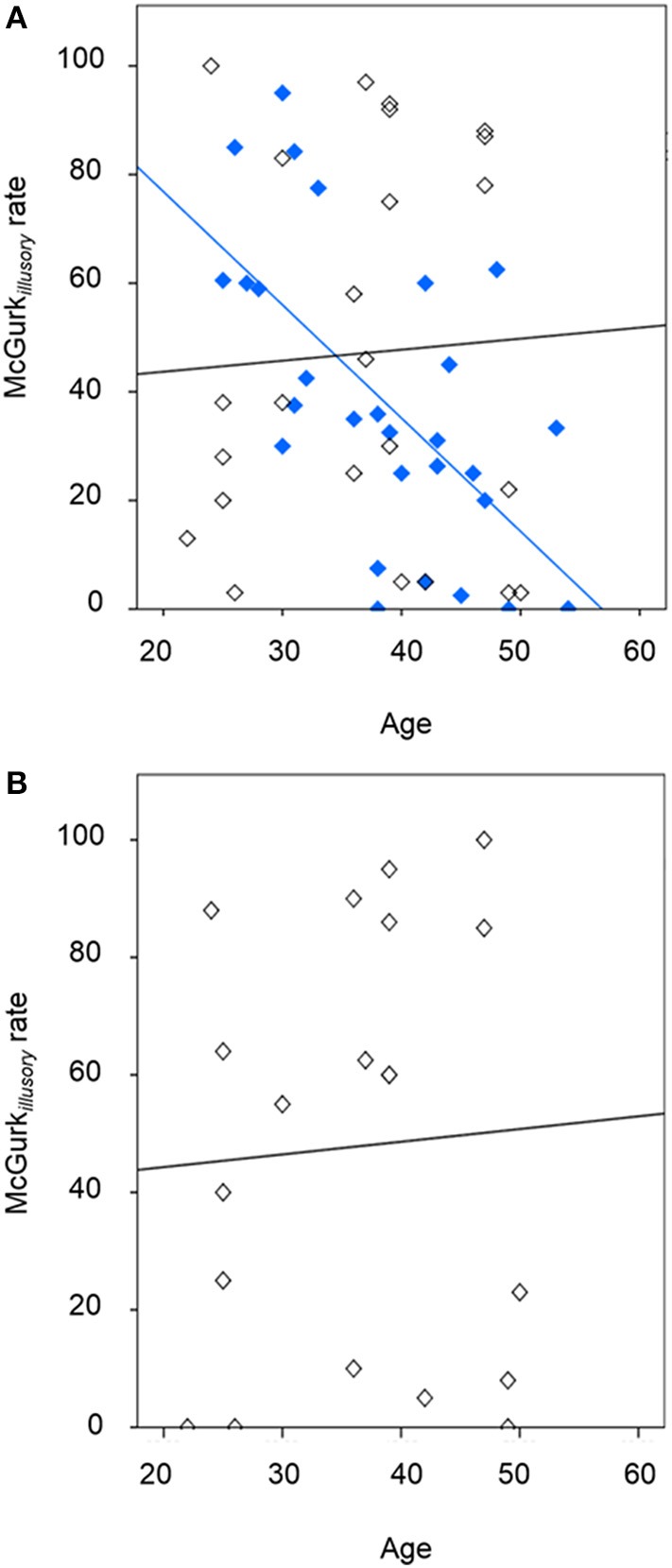
**The relationship between illusory perception and age, showing **(A)** the schizophrenia group (in blue; R^2^ of best-fit line: 0.414) and healthy individuals following placebo (in black; R^2^ of best-fit line: 0.003), and **(B)** the healthy individuals following amisulpride (in black; R^2^ of best-fit line: 0.003)**.

The McGurk*_*illusory*_* rate was significantly affected by study group [likelihood ratio χ^2^_(1, *N* = 53)_ = 4.91, *p* = 0.027], age [likelihood ratio χ^2^_(1, *N* = 53)_ = 4.55, *p* = 0.033; effect size, *r* = 0.29], and the interaction of these factors [likelihood ratio χ^2^_(1, *N* = 53)_ = 6.44, *p* = 0.011; effect size, *r* = 0.35]. Figure 1 depicts the between-group effect and Figure 2A illustrates the relationship between McGurk_*illusory*_ perception and age for each group. Age was inversely associated with McGurk_*illusory*_ rate in the schizophrenia group and accounted for 41.4% of related variance. In the healthy group, age accounted for just 0.3% of variance in the McGurk_*illusory*_ rate. Interestingly, there was a robust inverse relationship between illness duration and the McGurk_*illusory*_ rate in the schizophrenia group [unstandardised *B* = −0.02 ± 0.01, *t*_(28)_ = −4.14, *p* < 0.001; effect size, *r* = 0.62].

**Figure 3:**
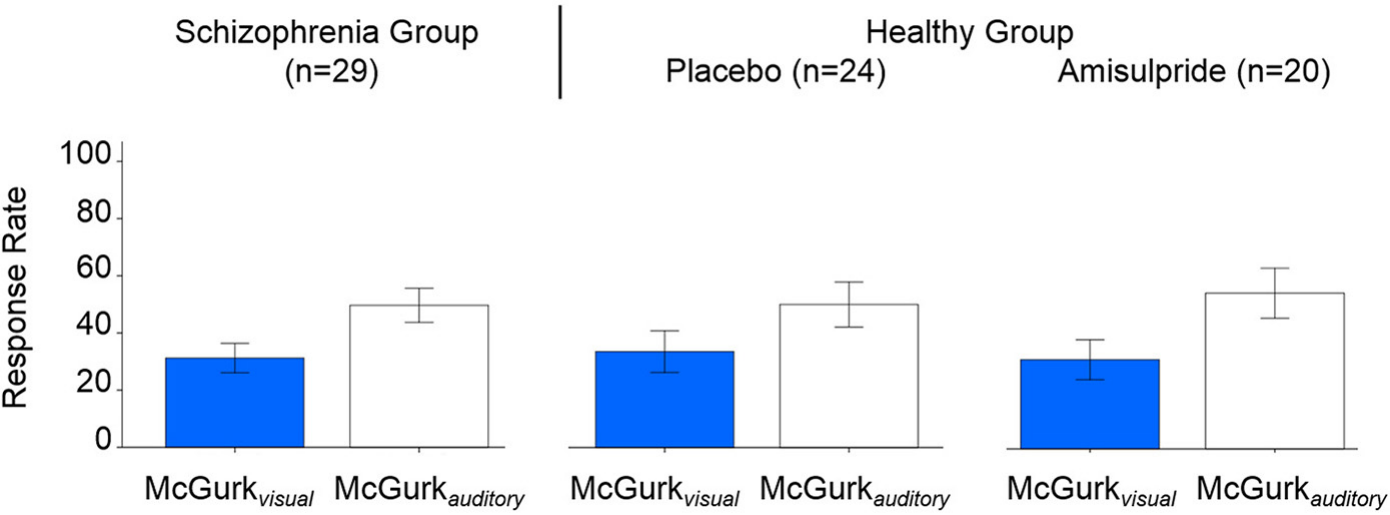
**Group-averaged McGurk_*visual*_ and McGurk_*auditory*_ rates, for the schizophrenia group (left), and the healthy group after placebo (center) and after amisulpride (right)**. Blue bars show McGurk_*visual*_ rates, white bars show McGurk_*auditory*_ rates. Error bars signify the standard errors of the mean.

**Figure 4:**
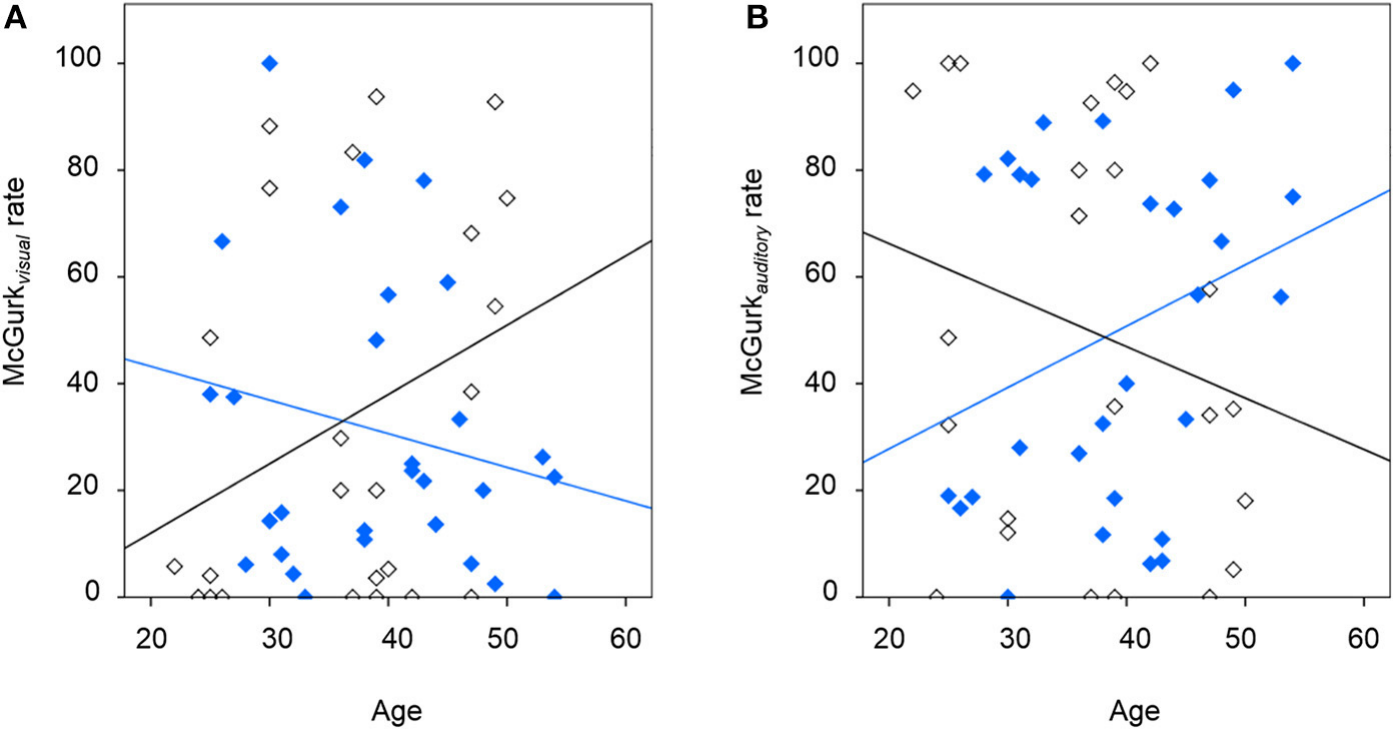
**The relationship between McGurk non-illusory perception and age, showing **(A)** findings for the McGurk_*visual*_ rate for the schizophrenia group (in blue; R^2^ of best-fit line: 0.035) and the significant relationship for healthy individuals following placebo (in black; R^2^ of best-fit line: 0.094), and **(B)** findings for the McGurk*_*auditory*_* rate for the schizophrenia group (in blue; R^2^ of best-fit line: 0.105) and healthy individuals following placebo (in black; R^2^ of best-fit line: 0.050)**.

Table 2 presents inferences related to the McGurk_*visual*_ and McGurk_*auditory*_ rates. Trends toward group effects were noted in both McGurk_*visual*_ and McGurk_*auditory*_ rates, and there was a significant group-by-age interaction in McGurk_*visual*_ rate. Figure 3 illustrates mean McGurk_*visual*_ and McGurk_*auditory*_ rates by group and Figure 4 depicts these rates with respect to age for each group.

**Table 2:** **Effects of group and age on the McGurk_*illusory*_, McGurk_*visual*_ and McGurk_*auditory*_ rates. Asterisks denote effects significant at an uncorrected α-level of 0.05**.

**Rate**	**Effect/Interaction**	**Inference**
McGurk_*illusory*_	Group	likelihood ratio χ^2^ = 4.91, *p* = 0.027*
	Age	likelihood ratio χ^2^ = 4.55, *p* = 0.033*
	Group-by-age	likelihood ratio χ^2^ = 6.44, *p* = 0.011*
McGurk_*visual*_	Group	likelihood ratio χ^2^ = 3.43, *p* = 0.064
	Age	likelihood ratio χ^2^ = 0.41, *p* = 0.525
	Group-by-Age	likelihood ratio χ^2^ = 3.85, *p* = 0.050*
McGurk_*auditory*_	Group	likelihood ratio χ^2^ = 3.67, *p* = 0.055
	Age	likelihood ratio χ^2^ = 0.06, *p* = 0.814
	Group-by-Age	likelihood ratio χ^2^ = 3.81, *p* = 0.051

Non-significant effects of age, group or their interaction were observed in perceptual performance during incongruent non-McGurk or control trials (Figure 1).

The McGurk_*illusory*_ rate did not significantly correlate with IQ over the full study sample (*r* = 0.150, *p* = 0.283) or for either study group individually (individuals with schizophrenia: *r* = 0.110, *p* = 0.569; healthy individuals: *r* = 0.086, *p* = 0.691). The McGurk_*illusory*_ rate was significantly negatively correlated with the McGurk_*auditory*_ rate in healthy individuals (*r* = −0.529, *p* = 0.009) but not individuals with schizophrenia (*r* = −0.299, *p* = 0.115). Non-significant correlations were observed between the McGurk_*illusory*_ rate and the McGurk_*visual*_ rate in either group.

No relationships between perceptual performance and symptomatology were significant after correction for multiple comparisons. However, a positive relationship significant at an uncorrected threshold was observed between the McGurk_*illusory*_ rate and PANSS negative scores (rho = 0.439, *p* = 0.022). There was also a trend toward individuals with higher PANSS positive scores exhibiting a reduced McGurk_*illusory*_ rate (rho = −0.324, *p* = 0.092).

Medication dosage was not significantly associated with any assessed measures of task performance.

### Modulation of audio-visual integration by amisulpride

Figure 1 displays group-averaged perceptual performance in the healthy individuals after placebo and after amisulpride. GLMs showed non-significant modulation of perceptual performance by drug condition, age or the interaction of these factors in healthy individuals for McGurk, incongruent non-McGurk, or control trials. Figure 2B depicts the relationship between the McGurk_*illusory*_ rate and age in the healthy group after amisulpride. While this relationship is not significant, concurrent inspection of Figures 2A,B demonstrates the within-subject stability of the McGurk effect.

Figure 3 shows the McGurk_*visual*_ and McGurk_*auditory*_ rates across drug conditions. Non-significant modulation of these rates was observed by drug or its interaction with age. However, age was found to significantly positively predict the McGurk_*visual*_ rate [*F*_(1, 20)_ = 4.93, *p* = 0.039; effect size, *r* = 0.44] in healthy individuals across both drug conditions.

### Findings related to metacognitive performance

Table 3 presents group-averaged confidence ratings in association with the experimental task. The ANOVAs investigating confidence ratings and their variation by trial type, study group (healthy/schizophrenia) and age found a significant main effect of trial type on mean overall confidence [Mauchly's ω = 0.096, Greenhouse-Geisser corrected *F*_(2, 53)_ = 5.33, *p* = 0.023; effect size, *r* = 0.30], and on mean confidence during optimal performance [Mauchly's ω = 0.096, Greenhouse-Geisser corrected *F*_(2, 53)_ = 4.98, *p* = 0.030; effect size, *r* = 0.29]. There was a trend toward a condition-by-group interaction on mean confidence during optimal performance [Greenhouse-Geisser corrected *F*_(2, 53)_ = 3.87, *p* = 0.054; effect size, *r* = 0.26]. No other main or interaction effects were significant.

**Table 3:** **Confidence ratings categorized by experimental condition and group**.

**Condition**	**Measure**	**Group**		
		**Schizophrenia**	**Healthy**	
			**Placebo**	**Amisulpride**
McGurk	Overall	6.91 ± 1.96 (29)	7.03 ± 1.78 (24)	7.55 ± 1.63 (20)*
	Optimal response	6.56 ± 2.29 (25)	6.06 ± 1.93 (24)	6.84 ± 2.08 (17)
	Other response	7.01 ± 2.01 (29)	7.14 ± 1.77 (23)	7.83 ± 1.62 (19)
Incongruent	Overall	8.15 ± 1.47 (29)	8.84 ± 0.93 (24)	8.89 ± 0.89 (20)
	Optimal response	8.21 ± 1.44 (29)	8.86 ± 0.93 (24)	8.89 ± 0.90 (20)
	Other response	4.84 ± 2.38 (14)	6.28 ± 4.94 (3)	5.30 ± 4.71 (3)
Control	Overall	8.19 ± 1.44 (29)	8.78 ± 0.93 (24)	8.88 ± 0.88 (20)
	Optimal Response	8.22 ± 1.44 (29)	8.79 ± 0.93 (24)	8.90 ± 0.88 (20)
	Other response	7.41 ± 2.16 (22)	7.05 ± 3.22 (10)	6.44 ± 2.84 (5)

Group-averaged mean value ± standard deviations. Bracketed values denote number of individuals contributing to mean value. Asterisks denote significant differences between drug conditions in healthy individuals as assessed via paired-samples t-tests. Independent samples t-tests revealed no significant between-group differences in confidence.

The ANOVAs investigating amisulpride-related modulation of confidence revealed no significant main or interaction effects. However, a paired-samples *t*-test revealed that amisulpride induced increased overall confidence ratings during perception of McGurk stimuli compared with perception of the same stimuli after placebo [*t*_(19)_ = 2.20, *p* = 0.041; effect size, *r* = 0.45]. Exploratory paired samples *t*-tests for the other experimental conditions revealed non-significant modulation by drug.

## Discussion

Principal findings of this behavioral study of audio-visual integration were: that individuals with schizophrenia were less likely to perceive the McGurk illusion than healthy controls; and that, in these individuals but not control subjects, age significantly and inversely predicted the rate of illusory experience. The latter finding, in limiting associations with age to the patient group, implies a role for illness chronicity. This is substantiated by the observation that illness duration robustly predicted rates of illusory perception. Overall, these findings are consistent with the proposal of a Bayesian-like probabilistic inference process being at work in the McGurk effect, modification of this process by experience, and disturbance to this system in schizophrenia.

Reduced susceptibility to the McGurk effect has been inconsistently reported in individuals with schizophrenia (Myslobodsky et al., 1992; Surguladze et al., 2001; Pearl et al., 2009; Szycik et al., 2009; Martin et al., 2013). Several studies reporting null findings were underpowered to detect effects of small magnitude on account of their small samples. The inconsistency in the literature feasibly reflects these small-to-moderate sample sizes, heterogeneity in the clinical phenotype of the disorder and between-study variation in the sensory stimuli presented. However, as considerable inter-subject variability exists in susceptibility to illusions (as alluded to by previous analyses investigating perception in subsets of responders and non-responders), definitively establishing whether susceptibility is affected in schizophrenia fundamentally depends on identifying and accounting for factors controlling this variability.

The current observation that individuals with schizophrenia were less likely to bind auditory and visual information may seem at first glance incompatible with the considerable literature that individuals with the disorder require greater temporal asynchrony between stimuli to dissociate them (Foucher et al., 2007; Giersch et al., 2009; Schmidt et al., 2011). However, the McGurk effect relies on implicit multisensory binding, while judgments of temporal asynchrony are precisely explicit. Differences in the preservation of these capabilities are expected in light of the independence of implicit and explicit judgments and previously-observed differences in their impairment in schizophrenia (Lalanne et al., 2012a,b). Furthermore, irrespective of the conceptual independence of these judgment types, reduced susceptibility to illusions and reduced performance in temporal asynchrony tasks can be reasonably interpreted as deviation from the behavior of an optimal observer.

Disturbances to multisensory perception have also been observed in relation to emotional processing in schizophrenia. de Gelder et al. (2003, 2005) found in emotion recognition tasks that individuals with schizophrenia were less likely than healthy controls to benefit from confirmatory vocal evidence when assessing facial expression but also exhibited exaggerated face effects when judging vocal emotion. This denotes non-conformity with the general principle that responses to ambiguous stimuli are more influenced by a second stimulus even when this stimulus is extraneous (Massaro, 1998). Interestingly, the same individuals showed inconsistent voice categorization in even the least challenging task conditions suggesting high-level auditory uncertainty. It is therefore a vital future objective to dissociate deficiencies in perceptual decision making from those directly related to multisensory integration.

In spite of the previous observation that varying attentional load does not impact proficiency of multisensory fusion (Vroomen et al., 2001), it is feasible that the observed between-group differences have in part an attentional foundation. Individuals with schizophrenia have been replicably shown to exhibit reduced visual focus on socially-important face regions such as the mouth and eyes (Loughland et al., 2002a,b); which could underlie their performance abnormalities in this task, in addition to other social deficits. Future work would therefore be greatly strengthened by the collection of eye-tracking measurements during task performance. In fact, significant associations between poor sensory integration and eye-tracking disorders have been previously reported in schizophrenia (Ross et al., 1998).

Age interestingly moderated audio-visual performance, predicting the rate at which individuals perceived the McGurk effect. However, while significant associations with age were noted as a whole-sample characteristic, they were evidently stronger in the schizophrenia group, which suggests that the age-related decrements observed in the schizophrenia group are attributable to long-term illness rather than the ageing process itself. This notion is strengthened by the highly-significant relationship between illness chronicity and the rate of illusory perception seen in these individuals. An alternative perspective is that reduced illusory perception in individuals that have been longer diagnosed with schizophrenia is attributable to progressive abnormalities in brain structure and function (van Haren et al., 2008), which in turn impair the systems on which multimodal integration is dependent. The cumulative role of other interacting factors such as long-term medication is also unclear, although medication dosage was not related to perceptual performance in the current investigation.

The current findings partially contradict those of Pearl et al. (2009), who, despite similarly reporting diminished susceptibility to the McGurk effect in individuals with schizophrenia, observed an alternative age effect: healthy adolescents experienced the illusion more frequently than healthy adults, but perception did not differ by age in their stratified schizophrenia group. Pearl et al.'s (2009) findings are in line with the modality-appropriateness hypothesis, which suggests that the weighting of sensory signals differs through life, presumably on account of sensory-neural degeneration in later adulthood (Gordon-Salant, 2005). The McGurk effect is seemingly contingent on an individual possessing requisite acuity of both visual and auditory processing. Inter-subject variation in sight and/or hearing capacity may underlie the differing susceptibility to illusory perception exhibited between healthy individuals; although this remains a speculative interpretation in the absence of explicit sense testing. The discrepancy between our healthy-sample findings and those of Pearl et al. (2009) may be partially attributable to the previous study recruiting individuals whose ages ranged beyond that of those included in the current sample, and who were therefore more likely to exhibit sensory decline. Furthermore, the mean illness duration of the current schizophrenia group (13.9 ± 8.7 years) was greater than that of the previous study (7.2 ± 6.2 years), which potentially enhanced our sensitivity to detect effects of illness chronicity.

Another, alternative relationship between age and susceptibility to the McGurk effect was observed by Setti et al. (2013), who found increased illusory perception in a sample of older adults (mean age: 65 years) compared with younger adults (mean age: 22 years) and interpreted this increase in terms of perceptual decline in the older group. The current findings are compatible with these observations, on account of the differences in age distribution between the study samples. However, the interpretation that McGurk illusory perception is more likely in individuals with deficient sensory performance is somewhat at odds with the view that it represents optimal function. Cienkowski and Carney (2002) also reported evidence of normal-to-enhanced McGurk effects in a small sample of older as compared with younger adults. This study elegantly measured their participants auditory (but not visual) capabilities but its conclusions are somewhat limited by a small sample size. The effects of age-related sensory decline on illusory perception remain to be definitively characterized.

Deficits in the formation and updating of expectations potentially underlie schizophrenia-related abnormalities in reward processing (Heinz and Schlagenhauf, 2010; White et al., 2013); sensorimotor predictive coding (Shergill et al., 2005, 2014); and wide-ranging features of perception (Phillips and Silverstein, 2013), including multisensory perception. The impact of expectations on all of these processes highlights their fundamental importance for adaptive behavior; and intimates pervasive difficulties for individuals in whom this system breaks down. Nevertheless, in light of the heterogeneity in the clinical presentation of schizophrenia, the dynamic nature of probabilistic inference and the numerous domains in which it has been found to be abnormal in schizophrenia, it remains a considerable challenge to isolate any specific putative disturbance in the disorder. Computational modeling offers the most viable current means of characterizing the relevant functional components. Investigation of specific inference-related parameters (for example, the temporal trade-off between existing and new evidence) offers a window on the clinical features of schizophrenia (Joyce et al., 2013). Similarly, such models may permit future dissociation of aberrances in unimodal perception and those of defective integration. However, this may be a false distinction in Bayesian terms (in some circumstances), since it is possible that a single pathophysiological abnormality could echo through the inherently hierarchical system impairing both processes.

A reduced McGurk_*illusory*_ rate, indexing an impairment to multisensory predictive inference, was expected in individuals with schizophrenia with prominent positive symptoms, given Bayesian accounts of these symptoms (Fletcher and Frith, 2009). However, while a trend toward this putative relationship was indeed observed, more robust was the positive association between negative symptoms and the McGurk_*illusory*_ rate. Impaired probabilistic inference can be reasonably hypothesized to underlie negative symptoms since continually encountering seemingly-unreliable sensory data could plausibly hinder decisions, render actions inconsequential and ultimately induce the paucities of thought and movement associated with negative symptoms. Paradoxically, in the current dataset individuals with prominent negative symptoms were more susceptible to the illusion. Thus, while the Bayesian account offers a compelling procedural explanation for multisensory integration (Seilheimer et al., 2014) and its abnormality in schizophrenia, alternative explanations should also be considered. For instance, dysconnectivity of brain structure, function, and mental experience have been diversely observed in schizophrenia (Fitzsimmons et al., 2013; Palaniyappan et al., 2013; White et al., 2013), and may fundamentally impair the inter-regional synchronization of activity upon which effective multisensory integration depends. Alternatively, impaired multisensory fusion in schizophrenia could potentially be attributable to modality-specific attentional deficits in these individuals.

The investigation of metacognitive appraisal of multisensory perception yielded consistent effects of experimental condition, with lower confidence ratings in association with the ambiguous illusion-eliciting stimuli as compared with control stimuli and a tendency for this effect to be less pronounced in individuals with schizophrenia than healthy individuals. This result can be interpreted as the control conditions proving more challenging for the individuals with schizophrenia; and healthy individuals exhibiting enhanced awareness of the intrinsic ambiguity of cue information. Nevertheless, main group effects were not significant. The relationship between confidence and dopamine potentially holds the key for understanding several core clinical features of schizophrenia. Delusional beliefs are confidently held without logical substantiation; it is possible that belief formation governed by phasic dopamine release is aberrant in schizophrenia due to dysregulated firing in association with conventionally non-salient events (Kapur, 2003); and further that the hyper-dopaminergic tonic dopamine activation produces the immutable, high-confidence associated these beliefs. Failure to appropriately update expectations could explain both the liberal acceptance thresholds seen during decision making in schizophrenia (Moritz et al., 2007), and cognitive predispositions such as the bias against disconfirmatory evidence (Moritz and Woodward, 2006; Woodward et al., 2007).

We report for the most part null effects of amisulpride administration on multisensory performance. This may reflect in part the relative timing of dosing and task performance. The initial T_*max*_ of amisulpride is associated with lower plasma concentration than the second (Kudris et al., 2011); it is therefore possible that the current investigation was not advantageously placed to investigate dopaminergic modulation of perception.

A limitation of the current protocol is the differential use of placebo conditions between the schizophrenia and healthy groups. While the concordance of our results with previous investigations of the McGurk effect in schizophrenia (de Gelder et al., 2005; Pearl et al., 2009) suggest that this is not the sole driver of our between-group differences, previous links between placebo responses and dopamine warrant discussion. Striatal dopamine has been shown to be positively associated with both anticipatory responses during reward learning (with the latter indices of the effects of Bayesian priors), and the level of placebo responses to preparations believed to have anti-nociceptive properties (Scott et al., 2007). Direct investigation of placebo effects on multisensory integration is therefore a worthwhile line of future research. In addition, it would be useful for future work on multisensory binding in schizophrenia to use Bayesian models to evaluate the effects of shifting stimulus onset asynchronies between auditory and visual information to ascertain the weighting of reciprocal effects of each modality on the other. A further potential limitation of this work is the imperfect between-group IQ matching. However, the WASI indexes current rather than premorbid IQ and the current sample exhibited similar (if slightly higher) scores to individuals with schizophrenia tested previously with the same scale (Weickert et al., 2000; Barrett et al., 2009). Together, these findings demonstrate the test's sensitivity to the cognitive deficits characteristic of the schizophrenic clinical phenotype. Furthermore, the control and patient groups were well matched in terms of age, sex and socioeconomic background, which serves to validate the recruitment procedures used.

While the focus of the current work is on dopamine, whose effects appear to enforce top-down adaptation of signaling, there is evidence that other neuromodulatory transmitters play complementary roles in Bayes-guided processing. Acetylcholine, for instance, has been recently shown to boost bottom-up signaling in predictable environmental conditions (Moran et al., 2013). In light of the inherent interdependence of these and other neurotransmitter systems, and the hierarchical and reciprocal nature of probabilistic inference (Friston and Kiebel, 2009; Feldman and Friston, 2010; Friston et al., 2012), determination of transmitter-specific computational effects and related interactions will also greatly improve our understanding of related cerebral mechanisms.

In summary, this work further demonstrates the utility of illusion-provoking scenarios for the investigation of information-processing abnormalities in schizophrenia (Dima et al., 2009; White and Shergill, 2012). In addition to confirming attenuated rates of illusory experience in the disorder, which are interpreted as an indication of sub-optimal inference, we present data on the subjective milieu surrounding perception of these ambiguous sensory situations. The incorporation of metacognitive appraisals provides a window on the dynamic effects of expectation not only on perception *per se* but also inferences drawn from and about it. While alternative explanations cannot be unequivocally discounted, our findings add to the flourishing literature suggesting that schizophrenic pathophysiology is usefully framed in Bayesian terms.

### Conflict of interest statement

The authors declare that the research was conducted in the absence of any commercial or financial relationships that could be construed as a potential conflict of interest.
